# Physical, Chemical and Biological Characteristics in Habitats of High and Low Presence of Anopheline Larvae in Western Kenya Highlands

**DOI:** 10.1371/journal.pone.0047975

**Published:** 2012-10-23

**Authors:** Bryson A. Ndenga, Jemimah A. Simbauni, Jenard P. Mbugi, Andrew K. Githeko

**Affiliations:** 1 Department of Zoological Sciences, Kenyatta University, Nairobi, Kenya; 2 Centre for Global Health Research, Kenya Medical Research Institute, Kisumu, Kenya; Swedish University of Agricultural Sciences, Sweden

## Abstract

**Background:**

Characteristics of aquatic habitats determine whether mosquitoes will oviposit, hatch, develop, pupate and successfully emerge into adults or not, thus influencing which mosquito species will occupy a habitat. This study determined whether physiochemical and biological characteristics differ between habitats with high and low presence of anopheline larvae.

**Methods:**

Physical, chemical and biological characteristics were evaluated in selected habitats twice per month within three highland valleys in western Kenya. Aquatic macro-organisms were sampled using a sweep-net. Colorimetric methods were used to determine levels of iron, phosphate, nitrate, ammonium and nitrite in water samples. Generalized Estimating Equations (GEE) was used to compare parameters between the two categories of anopheline presence.

**Results:**

Habitats with high anopheline presence had greater abundance of mosquito aquatic stages and tadpoles and two times more levels of nitrate in water, whereas habitats with low anopheline presence had wider biofilm cover and higher levels of iron in water.

**Conclusion:**

Habitats of high and low presence of anopheline larvae, which differed in a number of physical, chemical and biological characteristics, were identified in valleys within western Kenya highlands. Differences in habitat characteristics are critical in determining the number of anopheline larvae that will fully develop and emerge into adults.

## Background

Aquatic habitats are an important component of the process that results in malaria transmission. Mosquito life cycle processes including oviposition, larval development, pupation and emergence occur in aquatic habitats. These habitats are crucial in determining the types of malaria vectors present in an area, their abundance and also the population dynamics of emerging adult mosquitoes [1], [2]. Immature stages of malaria vectors prefer different habitat types [3]–[5]. These habitats differ in their physical, chemical and biological characteristics [6]. Therefore, understanding habitat bio-physicochemical characteristics, anopheline larval dynamics and productivity of adult malaria vectors can be useful in improving Larval Source Management (LSM) operations.

Malaria in the sub-Saharan Africa (SSA) is mainly transmitted by *Anopheles gambiae sensu lato* and *An. funestus*, which are known to breed in open sun-lit pools of water and relatively large permanent water bodies with vegetation, respectively [7]. However, these vectors have been found breeding in a great variety of aquatic habitats [8]–[17]. Several factors have been postulated in an attempt to explain why these vectors are present, abundant or their adults produced in large numbers in some habitats and not in others. These factors include oviposition behaviour of female mosquitoes [18], [19], physical, chemical and biological characteristics of habitats [8], [17], [20], [21], land cover and change in land use [3], [22], local climatic characteristics [23] and topography [24]–[26].

In most areas, it has been observed that only about a third to two thirds of all available habitats usually have anopheline larvae and only a few of these habitats produce a high number of adult vectors [4], [9], [20], [27]. Similar observations have also been made in three highland valleys of western Kenya (Ndenga *et al* unpublished observations). It was observed that anopheline early and late instar larvae were present in 37.7% and 17.6% of all the samples, respectively, that were made from late May to late August in 2008. Furthermore, it was observed that chances of finding anopheline larvae in some habitats were higher than in others. Therefore, the aim of this study was to determine whether physical, chemical and biological characteristics significantly differ between habitats of high and low anopheline presence over a one year period.

## Materials and Methods

### Study area

**Figure 1:**
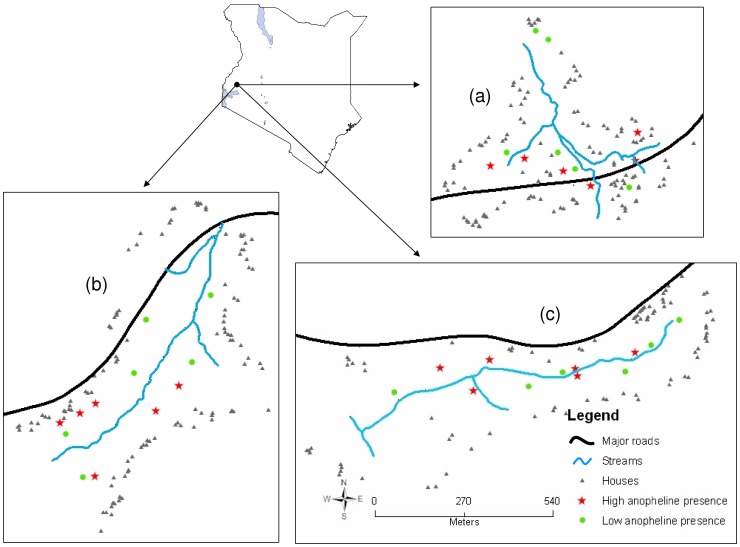
Map showing anopheline breeding habitats whose physical, chemical and biological characteristics were repeatedly evaluated for 12 consecutive months at Musilongo (a), Emutete (b) and Kezege (c).

The study was carried out in three valleys, namely, Musilongo (Universal Transverse Mercator (UTM) latitude 0.0208; longitude 34.6035; altitude 1500 meters above sea level (m.a.s.l.); area 0.16 km^2^), Emutete (latitude 0.0260; longitude 34.6358; altitude 1506 m.a.s.l.; area 0.24 km^2^) and Kezege (latitude 0.0264; longitude 34.6506; altitude 1545 m.a.s.l.; area 0.20 km^2^) within the highlands of western Kenya (Figure 1). These sites are located along the Luanda–Majengo Road in currently Vihiga County [5]. Subsistence farming is the main economic activity in these highly populated areas. This has resulted in reclamation of natural swamps, by digging open water drains, within these valley bottoms to create farms suitable for crop cultivation.

### Selection of habitats

A preliminary survey was done to determine the presence and absence of anopheline larvae in all aquatic habitats twice a month from late May to late August 2008 in the three study sites. A total of seven sampling visits were made in each habitat and counts of the number of times it had water and also anopheline larvae were made. Out of all the 786 individual habitats that were repeatedly sampled, 44 (5.6%) had anopheline larvae in all the seven visits whereas 85 (10.8%) had no anopheline larvae at all. A habitat that had anopheline larvae in all the seven sampling visits qualified to be selected in the category of habitats with high presence of anopheline larvae. On the other side, a habitat that did not have anopheline larvae in all of the seven sampling visits qualified to be selected in the category of habitats with low presence of anopheline larvae. Six habitats in each of these two categories within each of the three study areas were randomly selected for sampling. Overall, a total of 36 habitats were selected for the study (12 habitats in each study area (Figure 1).

### Sampling and characterization of habitats

Sampling of physical, chemical and biological characteristics in the selected habitats was done twice a month (second and fourth week of every month) for 12 consecutive months from September 2008 to August 2009. Since it was necessary to capture characteristics of all the six habitats in each category and each site at every sampling visit, in case a habitat was dry at the time of sampling, it was substituted with another one from the same category. This implies that other habitat characteristics except habitat stability, which is an important factor in determining habitat productivity [26], were considered in the scope of this study. Each selected habitat was sampled using a sweep net [5] to determine the number of mosquito larvae, pupae and other aquatic macro-organisms present. The sweep net was made of a fine cloth which enabled the collection of first instar larvae. This sweep net measured 40 cm long, 15 cm wide and 30 cm high. It was gently dragged along the entire water surface, at an angle of 45°, at least three times until no more macro-organisms were collected. Sweeping was done at the edges of large habitats where larvae and pupae aggregate [28]. Information that was collected on each visit included date of sampling, name of the study site, length and width (in cm) of water surface area, water depth and width (in cm, using an aluminium meter rule) at three different points and length (using a 30 m measuring tape), water flow (described as stagnant or flowing) and water temperature (°C, using an industrial thermometer). Cover (in percentage of water surface area) of filamentous algae, emergent vegetation and visible surface biofilm that formed an oil-like layer on the water surface was visually estimated. Height of three different emergent plants was measured (in cm using an aluminium meter rule) from the water level. Levels of pH in water were measured using a hand-held electronic unit (pH Test Pen PHT-01 ATC, Voltcraft®, 92242 Hirschau, Germany). Water conductivity in micro Siemens per cm (µS/cm) was measured using a hand-held electronic unit (HI 98311–HI 98312 Waterproof EC/TDS/Temperature Testers, Hanna Instruments, Maurituis). Water chemical composition analyses for iron (in mg/l Fe), phosphate (in mg/l PO_4_^3−^), nitrate (in mg/l NO_3_^−^), ammonium (in mg/l NH_4_^+^) and nitrite (in mg/l NO_2_^−^) were measured using colorimetric methods (Merck KGaA, 64271 Darmstadt, Germany). Testing of water samples was done on site immediately after collection. Counts of anopheline and culicine early (1^st^ and 2^nd^) instar larvae and late (3^rd^ and 4^th^) instar larvae, mosquito pupae, water beetles (Family Dytiscidae), dragonfly nymphs (Suborder Anisoptera) and damselfly nymphs (Suborder Zygoptera), water scorpions (Family Nepidae), backswimmers (Family Notonectidae), creeping water bugs (Family Naucoridae) and water striders (Family Gerridae), small fishes common in streams (Family Poeciliidae) and tadpoles (Family Pipidae) were recorded. A maximum of three anopheline larvae per sample were collected in 20 ml vials with a screw cork loosely tightened and half filled with water from the respective habitat and transported in a cooler box to the insectaries 25 km away at Kenya Medical Research Institute (KEMRI) Centre for Global Health Research (CGHR) in Kisumu located at Kisian. In the laboratory, the anopheline larvae were examined under a compound microscope at X40 magnification for the presence/absence of *Coelomomyces* species and *Vorticella* species.

### Ethical considerations

This study was approved by KEMRI/National Ethical Review Committee (SSC No. 1328). Verbal consent to access compounds and farms was obtained from local leaders and residents during village administrative meetings in each of the study areas.

### Data analysis

Abundance of aquatic macro-organisms was defined as the number of individuals per metre square of water surface area. Non-mosquito aquatic arthropod macro-organisms were grouped into three categories, namely: Odonata, Coleoptera and Heteroptera. Average water depth and height of emergent plants were calculated per sample. Generalized Estimating Equations (GEE) was used to calculate means (95% Confidence Interval) and test for statistical differences in the parameters that were repeatedly measured between the habitats of low and high anopheline presence. Correlations of anopheline late instar larvae and significantly different habitat parameters were determined using Pearson correlation coefficient. Data was analysed using SPSS version 16.

## Results

### Larval habitats sampled

A total of 864 samples (432 in the high and also 432 in the low habitats of anopheline presence) were made in the selected habitats during the entire study period. Of these habitats, 740 (85.6%) were open drains, 77 (8.9%) burrow pits, 43 (5.0%) cultivated swamps, 2 (0.2%) natural swamps, 1 (0.1%) river fringe and 1 (0.1%) puddle. Out of all the habitats sampled, 766 (88.7%) were located within farmlands, 84 (9.7%) in grasslands and 14 (1.6%) were under *Eucalyptus* tree canopy. Overall, 859 (99.4%) of the habitats sampled originated from human related activities whereas 5 (0.6%) occurred naturally. Water was stagnant in 404 (46.8%) habitats and flowing in 460 (53.2%) at the time of sampling. In total, 28 (75.9%) habitats out of the selected 36 were sampled in 20–24 surveys; 20 (55.6%) of them were sampled in all the 24 surveys (Table 1).

**Table 1:** Number of times habitats were sampled in all the 24 surveys

Surveys	Habitats	Samples (%)
1	8	8 (0.9)
2	5	10 (1.2)
4	3	12 (1.4)
5	3	15 (1.7)
7	2	14 (1.6)
9	1	9 (1.0)
11	2	22 (2.5)
13	1	13 (1.5)
15	1	15 (1.7)
17	2	34 (3.9)
18	1	18 (2.1)
19	2	38 (4.4)
20	1	20 (2.3)
21	2	42 (4.9)
22	1	22 (2.5)
23	4	92 (10.6)
24	20	480 (55.6)
	59	864 (100)

### Aquatic fauna sampled

Mosquitoes sampled included 11,705 anopheline early instar larvae, 1,072 anopheline late instar larvae, 4,698 culicine early instar larvae, 969 culicine late instar larvae and 1,559 pupae. Other macro-organisms sampled included 285 Coleoptera (water beetles), 708 Odonata (dragonfly and damselfly nymphs), 1,516 Heteroptera (water scorpions, backswimmers, creeping water bugs, and water striders), 517 fishes and 4,943 tadpoles. Among 853 anopheline larvae that were examined under a microscope, 264 (30.9%) had *Vorticella* species and none of them was infested with *Coelomomyces* species.

### Comparison of habitat characteristics

**Figure 2:**
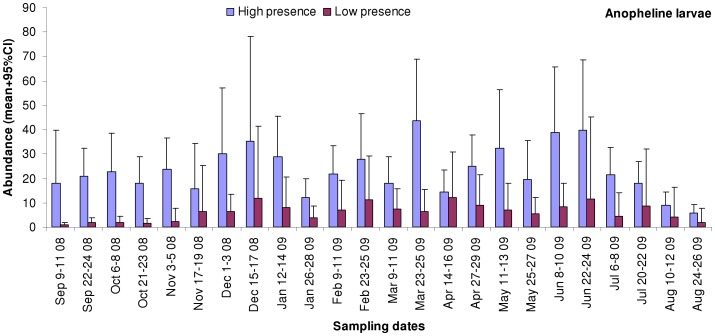
Dynamics of anopheline larvae (mean + upper 95%CI) in the habitats of high and low anopheline presence in each of the 24 surveys.

Out of the 404 habitats with stagnant water, 211 (52%) were from the habitats of low anopheline presence whereas 193 (48%) were from the habitats of high anopheline presence. Out of 27 parameters that were measured in the selected habitats, 10 of them were significantly different between habitats of low and high anopheline presence (Table 2). Habitats of high anopheline presence were associated with significantly higher number of habitats with anopheline larvae and of anopheline larvae in habitats; higher abundance of anopheline early and late instar larvae, culicine late instar larvae, mosquito pupae and tadpoles and two times more levels of nitrate compared to habitats of low anopheline presence (Table 2). On the other hand, habitats of low anopheline presence had significantly wider biofilm cover and higher levels of iron in water than habitats of high anopheline presence (Table 2). There was no statistical difference in the abundance of culicine early instar larvae; water depth; water surface area; water temperature; pH; conductivity; levels of phosphate, ammonium and nitrite; percentage cover of filamentous algae and emergent plants; height of emergent plants; abundance of Odonata, Coleoptera, Heteroptera and fishes and the proportion of anopheline larvae with *Vorticella* species between these two categories of anopheline presence (Table 2). Dynamics of the abundance of anopheline larvae (Figure 2) indicate that they were consistently and significantly higher (GEE, *P*<0.001) in the habitats of high anopheline presence than in the low ones in all the 24 surveys. The highest difference in abundance between these two categories was 16.8-fold in the September 9–11 2008 survey, whereas the lowest was 1.2-fold in the April 14–16 2009 survey.

**Table 2:** A contrast of physical, chemical and biological characteristics between habitats of high and low presence of anopheline larvae.

Descriptive	LowPresenceMean (CI)	HighPresenceMean (CI)	*P* value	Foldincrease
Samples	432	432		1.0
Habitats with anopheline larvae	199 (46.1%)	381 (88.2%)	<0.001	1.9
Number of anopheline larvae sampled	6.3 (3.1–12.6)	23.3 (17.6–30.9)	<0.001	3.7
**Mosquito abundance/m^2^**				
Anopheline early instar larvae	1.5 (0.7–3.3)	9.5 (3.9–23.3)	0.002	6.5
Anopheline late instar larvae	0.11 (0.06–0.20)	0.53 (0.34–0.82)	<0.001	4.8
Culicine early instar larvae	2.5 (0.8–8.3)	4.0 (1.2–13.1)	0.590	1.6
Culicine late instar larvae	0.2 (0.1–0.3)	1.1 (0.4–3.4)	0.001	6.6
Mosquito pupae	0.3 (0.2–0.4)	1.2 (0.5–2.6)	0.002	4.1
**Habitat characteristics**				
Water depth (cm)	6.1 (4.8–7.9)	8.8 (6.1–12.9)	0.110	1.4
Water surface area (m^2^)	9.2 (6.6–13.0)	7.8 (5.5–11.0)	0.526	0.8
Water temperature (°C)	23.2 (22.4–24.0)	23.4 (22.6–24.2)	0.545	1.0
pH	6.81 (6.76–6.87)	6.81 (6.74–6.88)	0.946	1.0
Conductivity (µS/cm)	69.7 (56.5–85.9)	58.1 (48.4–69.7)	0.122	0.8
Iron (mg/l Fe)	0.6 (0.5–0.7)	0.4 (0.3–0.5)	0.001	0.7
Phosphate (mg/l PO_4_^3−^)	0.014 (0.012–0.017)	0.018 (0.013–0.027)	0.247	1.3
Nitrate (mg/l NO_3_^−^)	1.8 (1.4–2.4)	3.6 (2.5–5.2)	0.005	2.0
Ammonium (mg/l NH_4_^+^)	0.25 (0.21–0.30)	0.20 (0.16–0.25)	0.143	0.8
Nitrite (mg/l NO_2_^−^)	0.015 (0.012–0.019)	0.019 (0.010–0.038)	0.473	1.3
Filamentous algae cover (%)	5.4 (3.6–8.3)	8.9 (6.1–12.8)	0.063	1.6
Biofilm cover (%)	37.2 (30.2–45.8)	10.0 (7.3–13.6)	<0.001	0.3
Emergent plant cover (%)	32.5 (25.4–41.7)	27.6 (20.7–36.9)	0.458	0.8
Emergent plant height (cm)	44.4 (36.8–53.6)	50.8 (43.8–58.8)	0.246	1.1
Odonata/m^2^	0.2 (0.1–0.3)	0.3 (0.1–0.5)	0.216	1.5
Coleoptera/m^2^	0.2 (0.1–1.0)	0.2 (0.1–0.9)	0.954	1.1
Heteroptera/m^2^	0.6 (0.2–1.6)	0.4 (0.2–0.6)	0.397	0.6
Fishes/m^2^	0.08 (0.04–0.15)	0.09 (0.04–0.23)	0.727	1.2
Tadpoles/m^2^	0.9 (0.6–1.4)	5.5 (1.9–15.9)	0.001	6.1
Anopheline larvae with *Vorticella* species (%)	23.8 (11.9–47.8)	34.0 (22.2–52.1)	0.301	1.4

CI = 95% Confidence Interval

### Correlations of significantly different habitat parameters

There was a positive and significant correlation between the abundance of anopheline late instar larvae and tadpoles; positive but non-significant correlation with levels of nitrate in water and negative but significant correlation with both levels of iron in water and biofilm cover on water surface (Table 3). There was no correlation between abundance of tadpoles in habitats and biofilm cover on water surface. Significant but negative correlation existed between levels of nitrate in water and biofilm cover on water surface. There was a positive and significant correlation between the levels of iron in water and bifilm cover on water surface. Fifteen habitats with highest levels of nitrate of 10 and 20 mg/l NO3- had a mean of 12.0 (11.3–12.7); 3 (20%) of them were from habitats of low anopheline presence, whereas 12 (80%) were from habitats of high anopheline presence. Fifty one habitats with highest levels of iron of 0.8 and 1.0 mg/l Fe had a mean of 0.51 (0.48–0.55); 34 (66.7%) of them were from habitats of low anopheline presence, whereas 17 (33.3%) were from habitats of high anopheline presence.

**Table 3:** Correlations of anopheline late instar larvae and significantly different habitat parameters.

Variable 1	Variable 2	Correlation	t-test	df	*P*-value
Anopheline late instar larvae	Tadpoles	0.121	3.574	862	0.0004
Anopheline late instar larvae	Nitrate	0.102	1.375	178	0.1709
Anopheline late instar larvae	Iron	−0.246	−3.124	152	0.0021
Anopheline late instar larvae	Biofilm cover (%)	−0.157	−4.654	862	3.78E-06
Tadpoles	Biofilm cover (%)	0.0003	0.010	862	0.9921
Nitrate	Biofilm cover (%)	−0.270	−3.748	178	0.0002
Iron	Biofilm cover (%)	0.254	3.243	152	0.0015

## Discussion

This study has demonstrated that higher presence and abundance of anopheline larvae, culicine late instar larvae, mosquito pupae and tadpoles and two times more levels of nitrate were associated with habitats of high anopheline presence. The fact that the presence and abundance of all mosquito aquatic stages remained consistently and significantly higher in habitats of high anopheline presence than in those of low presence in a one year period demonstrates the existence of habitats with extreme anopheline occupancy within valley bottoms of western Kenya highlands. Presence of the anopheline late instar larvae in habitats is used as a proxy measure for habitat productivity of malaria vectors [27], [29]. However, in some studies only the presence/absence of anopheline larvae is used [10], [11], [17], [26]. In both cases, there is the risk of overestimating the productivity of malaria vectors from habitats due to the presence of other *Anopheles* species [3], [5]. Anopheline larvae have been found breeding in a wide range of aquatic habitats. These habitats include: natural swamps, cultivated swamps, river fringes, puddles, open drains and burrow pits [5]; habitats located in farmlands, forested areas and in swampy places [3]; active and abandoned fish ponds [12]; rice paddies [13], [30]; permanent and semi-permanent habitats [10]; tree holes [31]; organically polluted habitats [14]; unused swimming pools [15]; drainage channels, hoof prints and tyre tracks [4], [22] and brick making sites [11]. Presence of anopheline larvae in these habitats may be as a result of hatching of the eggs oviposited in them [18], [19] or because eggs/larvae were transported in the habitats, most likely by flowing water. However, the development of larvae to emerge into adult mosquitoes is determined by the physical, chemical and biological characteristics of habitats [8], [17], [20], [21]. Therefore, it is important to determine whether anopheline larvae in habitats develop to maturity and successfully emerge into adults or not. This can be achieved by carrying out longitudinal studies in selected sentinel habitats. A challenge to such approach would be the possibility of characteristics changing in individual habitats with time as most of aquatic habitats in this area originate from human related activities [3], [5]. This may impact LSM operations as unpredictable habitats may be difficult to target [32].

Abundance of tadpoles in habitats of high anopheline presence is due to the fact that they predate less on anopheline larvae [33], [34]. Marten and others [35] made similar observations in *An. albimanus* larval habitats in the Pacific region of Colombia. However, contrary to this finding, Munga and others [36] demonstrated in their experiments that caged *An. gambiae* mosquitoes laid few eggs in water conditioned with tadpoles. This avoidance behaviour may result to fewer larvae in habitats infested with tadpoles. A number of studies have failed to associate nitrate with high abundance of anopheline larvae in habitats [6], [24], [37], [38] as it was observed in this study. Furthermore, Mwangangi and others [39] did not find a significant association between nitrate and wing length which was used as an indicator of mosquito body size of *An. gambiae s.s*. This presents the need for more work to be done in order to establish the role nitrate plays in the breeding of anopheline larvae.

Wide biofilm cover on water surface area in habitats and high levels of iron in water were associated with habitats of low anopheline presence. This biofilm is of the floating type which develops at the water-air-interface forming an oily-like continuous layer [40]. Although it consists of numerous types of micro-organisms that may contain important food sources for mosquito larvae [41], wide layers on water surface may lead to suffocation of mosquito larvae. This in the long run may result to reduction in the presence and abundance of mosquito larvae in habitats. Whereas the abundance of tadpoles in habitats remained the same regardless the water surface area covered by biofilm, there was a decrease in biofilm cover with increase in levels of nitrate in water. However, water surface area covered by biofilm increased with increase of iron levels in water, but abundance of anopheline late instar larvae in the habitats decreased with their increase. High content of iron recorded in the habitats of low anopheline presence indicates that it is detrimental to their breeding. In their study, Obsomer and others [42] did not find any influence of iron on the breeding of *An. bamaii* in aquatic habitats. However, Kankaew [43] reported some association between ferric iron and the presence of anopheline larvae in habitats.

In the process of measuring physical, chemical and biological characteristics in all the six habitats in each category and each site at every sampling visit, over a half of the initially selected habitats were sampled in all the surveys whereas the rest dried up at least once. These ones were substituted by others but form their respective categories. Since the initial six habitats were randomly selected from habitats that met the selection criterion for either of the category and had equal chances of being selected, mixing of habitats to substitute the dried ones was not of concern. However, we acknowledge that variability of the parameters that were measured with time would be smaller within same habitats than among several habitats. Capturing such variability was very important in this study in order to describe well the category characteristics rather than individual habitat ones.

Although LSM against malaria vectors using larvicides is traditionally done in all aquatic habitats [16], [44], findings of this study show that there are consistently some habitats of high and low presence and abundance of anopheline larvae. In the recent past, there has been increasingly renewed interest that advocates for the development of tools that can be used to target malaria vector control in aquatic stages [16], [45], [46], and a call for an in-depth revaluation of LSM operations. This is because only habitats that produce significantly higher adult vectors may eventually contribute towards malaria transmission; hence ability to identify them may be important in implementing targeted LSM in space and/or time [4], [45]. Therefore, habitat characteristic differences identified in this study can be used to further explore practical and operational ways in which LSM can be targeted against malaria vectors. Such tools should be easy to use by field teams to clearly and effectively identify the most productive individual habitats for malaria vectors to be targeted during LSM operations. This is very important in order to overcome the question to whether such knowledge can be translated into successful LSM operations or not [32].

## Conclusion

This study has generated additional information by contrasting physical, chemical and biological characteristics between habitats of high anopheline presence and those of low presence. Habitats with high anopheline presence had greater abundance of mosquito aquatic stages and tadpoles and two times more levels of nitrate in water, whereas habitats with low anopheline presence had wider biofilm cover and higher levels of iron in water. Further investigations need to be done to evaluate which of these characteristic differences can be put to practical use to identify habitats to be targeted during LSM operations against malaria vectors.
